# “Chickens Are a Lot Smarter than I Originally Thought”: Changes in Student Attitudes to Chickens Following a Chicken Training Class

**DOI:** 10.3390/ani5030386

**Published:** 2015-08-21

**Authors:** Susan J. Hazel, Lisel O’Dwyer, Terry Ryan

**Affiliations:** 1 School of Animal & Veterinary Sciences, Faculty of Sciences, University of Adelaide, Adelaide 5371, Australia; 2 Australian Population and Migration Research Centre, Faculty of Humanities and Social Sciences, University of Adelaide, Adelaide 5005, Australia; E-Mail: lisel.odwyer@adelaide.edu.au; 3 Legacy Canine Behavior & Training, Inc, Sequim, WA 98382, USA; E-Mail: terry@legacycanine.com

**Keywords:** clicker training, practical classes, learning theory, animal sentience, attitudes towards animals

## Abstract

**Simple Summary:**

Our attitudes to animals are linked to our beliefs about their cognitive abilities, such as intelligence and capacity to experience emotional states. In this study, undergraduate students were surveyed on their attitudes to chickens pre- and post- a practical class in which they learnt to clicker train chickens. Students were more likely to agree that chickens are intelligent and easy to teach tricks to, and that chickens feel emotions such as boredom, frustration and happiness, following the practical class. Similar workshops may be an effective method to improve animal training skills, and promote more positive attitudes to specific animal species.

**Abstract:**

A practical class using clicker training of chickens to apply knowledge of how animals learn and practice skills in animal training was added to an undergraduate course. Since attitudes to animals are related to their perceived intelligence, surveys of student attitudes were completed pre- and post- the practical class, to determine if (1) the practical class changed students’ attitudes to chickens and their ability to experience affective states, and (2) any changes were related to previous contact with chickens, training experience or gender. In the post- *versus* pre-surveys, students agreed more that chickens are easy to teach tricks to, are intelligent, and have individual personalities and disagreed more that they are difficult to train and are slow learners. Following the class, they were more likely to believe chickens experience boredom, frustration and happiness. Females rated the intelligence and ability to experience affective states in chickens more highly than males, although there were shifts in attitude in both genders. This study demonstrated shifts in attitudes following a practical class teaching clicker training in chickens. Similar practical classes may provide an effective method of teaching animal training skills and promoting more positive attitudes to animals.

## 1. Introduction

Animals that are perceived to be closer cognitively to humans are considered more positively by humans than those that are not [1]. More positive attitudes to animals may include a greater consideration of their welfare, an important factor since Gandhi has stated “The greatness of a nation and its moral progress can be judged by the way its animals are treated”. Through training animals, people may become more aware of the cognitive abilities of animals, which may in turn lead to a more positive attitude to their care and treatment [2]. However, no studies to date have analyzed if training animals does in fact lead to a greater recognition of their cognitive abilities. Another important reason for theoretical and practical knowledge of animal training is to avoid using punishment-based training methods and human frustration when working with animals. This can also irrevocably damage the human–animal bond, for example, use of positive punishment or negative reinforcement in dogs has been associated with increased risk of aggression to both people and other dogs [3,4].

Understanding how animals learn is particularly important for undergraduate disciplines, such as animal and veterinary science, in which graduates are likely to work with animals. Even without explicitly training animals, in every interaction a person has with an animal the animal is learning. Thus, knowledge of how they learn can help even with routine husbandry procedures to make it lower stress both for the animals and for the person. Learning theory provides relatively simple principles for training animals. However, practice is needed in applying these principles using real animals for students to be competent trainers. A problem is that in large classes, the choice of an animal species depends on availability, facilities and safety within a teaching environment. In 2007, two authors (Lisel O’Dwyer and Susan J. Hazel) ran a practical class for undergraduate students using horses, but not all students were competent at handling horses, and with them being a large and relatively expensive species, it was not possible to make enough animals available for all students to be actively involved. Dogs are easier to handle, but it is difficult to find sufficient numbers of dog owners who do not mind what novice students may teach their pets.

Practical classes with zoo animals have been used to teach students operant conditioning in an experimental psychology course [5]. Formal and informal student responses from the 15 students were extremely positive, and the authors concluded it was an effective class to teach the principles of operant conditioning in an applied setting. However, use of zoo animals would not be a practical option for class sizes of more than 100. An alternative species which is small, easy to handle, and learns quickly is the chicken [6,7,8]. Chickens would also be an attractive species to answer the question if training an animal influences a person’s attitude to them, as people may not see them as individual animals, instead associating them with masses of thousands in battery cages for the production of eggs, or in large broiler sheds for production of chicken meat. They have also been viewed in popular culture as having limited intelligence or character [9,10]. Generally, most urban dwellers have had little or no contact with chickens while rural dwellers who might keep chickens for eggs, meat or disposal of scraps do not train them.

The Brelands’ chicken shows in the 1950s in the USA were highly popular, demonstrating that chickens could be trained to perform complex and reliable behaviors using the principles developed by Burrhus Skinner [11], with chickens playing the piano and tap dancing in costume. More recently in the early 1990s, classes involving training chickens were developed by Terry Ryan to develop training skills in people wanting to train other species, such as dogs [6].

Chickens have other advantages over most other species when used for teaching students how to train an animal. Training a novel species helps people think of training from the animal’s point of view. Unlike chickens, dogs are generally willing to please and forgiving of human transgressions. Chickens are not as forgiving and leave little room for inappropriate methods. They do what is reinforcing for them to do, without concern for human approval [6]. Aversive training methods are especially counterproductive with this highly reactive species. Conversely, the type of relationship engendered with positive reinforcement using clicker training is mutually beneficial: the chicken thinks she is training the person to give food; the person thinks he or she is training the chicken to perform desirable behaviors. It is a win-win type of training that would greatly enhance the human–animal bond if employed with all species [6]. Chickens are also safely and easily handled and housed in sufficient numbers to allow all students sufficient “hands-on” experience.

In 2012, two authors (SH and LOD) attended a local workshop on training chickens using clicker training run by Terry Ryan [6]. Clicker training is positive reinforcement following a clicking sound which acts as a bridge, as popularized by Karen Pryor [12]. The bridge in operant conditioning is a secondary reinforcer; the animal learns its meaning as a feedback signal by the trainer pairing it with a primary reinforcer, such as food. The bridge provides almost instant feedback on what the animal is doing at that time, allowing time for the primary reinforcement (e.g., food) to then be delivered. Participation in the two-day workshop enabled preparation of a 2-h practical class clicker training chickens to undergraduate students, run for the first time in 2012.

Verbal feedback from students was immediately positive on what they learnt and how much fun the classes were. In addition, informal written feedback on the classes indicated that student attitudes to chickens changed following the class (*“I didn’t think chickens were that intelligent”; “I like the fact that chickens are smart, not just here to eat and lay eggs!”; “She was so smart. I really like chickens & now so much more, lots of respect for smart animals.”).* The present study was designed to further investigate the possible changes in undergraduate student attitudes to chickens, with the specific aims to determine if:

- a practical class involving clicker training chickens changes students’ attitudes toward chickens and their ability to experience affective states;
- changes in attitude are related to previous contact with chickens, training experience, or gender.

## 2. Methods

### 2.1. Student Participants

Students enrolled in the first year course “ANIML SC 1016RW/1018RW Principles in Animal Behaviour, Welfare & Ethics” participated in the study. This included Animal Science BSc (*n* = 83) and Veterinary Bioscience (*n* = 39) cohorts. The curriculum of the course includes a history of the study of animal behavior, learning theory, animal sentience, concepts of animal welfare, and animal ethics and is coordinated and largely taught by the first author (Susan J. Hazel). In the first eight weeks of the course, a 2-h practical class is offered on an eight week rotation (*i.e.*, the same practical classes are run each week with the students rotating through the different classes). This occurred between 1 August and 30 September 2013. A practical class teaching clicker training in chickens was one of these eight practical classes.

Students completed the surveys during their initial lecture in the course on the first day (pre-survey) and then following their completion of the chicken clicker training practical class (post-survey). This was between Weeks 1–8 of semester, meaning students came to the practical classes having covered varying parts of the curriculum (see Table 1). The learning theory module included non-associative learning (habituation and sensitization) and associative learning (classical and operant or instrumental conditioning). The instrumental conditioning included the four quadrants of conditioning; reinforcers; timing; reinforcement schedules; extinction; shaping; and features of good animal trainers. Since learning theory was not taught until the third week of the course, students who did clicker training in the first two weeks had not yet covered the material formally, although the reading materials for the Team-Based Learning (TBL) were made available from the first week of semester. 

**Table 1:** Curriculum covered in the first eight weeks of semester in “Principles in Animal Behaviour, Welfare & Ethics”.

Week	Topics Covered
1	Introduction to the course; A history of the study of animal behavior; Tinbergen’s hypotheses
2	Ethograms; Nature and Nurture; Behaviors of major species
3	Learning theory (TBL); Social behavior
4	Affective states, sentience and cognition
5	Animal Welfare (TBL); Welfare in zoo animals
6	Welfare in laboratory animals; Physiological measures of animal welfare (TBL)
7	Welfare in pigs; Welfare in fish
8	Methods used in animal slaughter; Behavioral measures of animal welfare (TBL)

TBL: Team-based Learning.

### 2.2. Chicken Clicker Training Practical Class

At the time of the practical classes, the 16 subject chickens (Hyline Brown Layers) were approximately 36 weeks of age. They were housed in a biosecurity unit in single tier cages (545 cm^2^/bird) and at the conclusion of the classes were re-homed in small free range holdings.

Each class was modified on a “Poultry in Motion” workshop using clicker training of chickens and run by Terry Ryan in July 2012 in South Australia [6]. Only minor modifications were made from the first running of the practical class in 2012.

**Figure 1:**
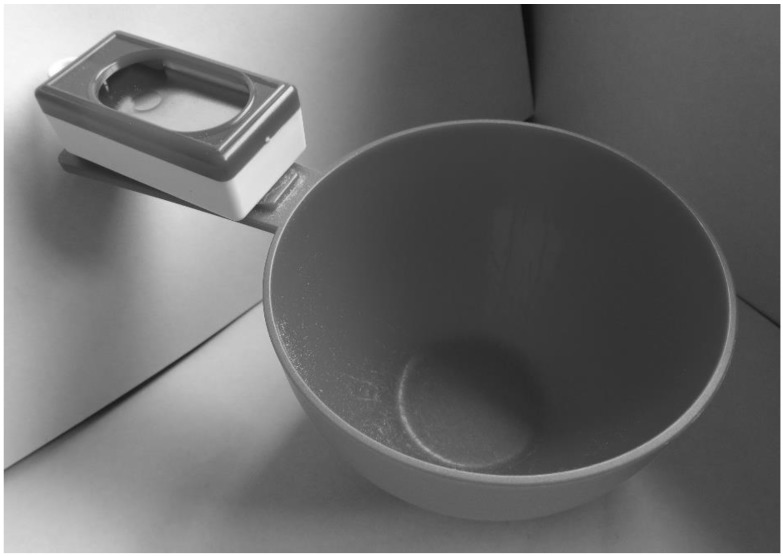
Photo of the plastic cup with clicker taped to the handle used in the clicker training practical classes.

The materials used in the practical classes included:

- A 250 mL plastic cup, with a clicker attached to the handle of the cup (Figure 1);
- Laminated coloured paper circles approximately 5 cm in diameter. Colours included red, green, purple and yellow;
- A laminated large black circle approximately 7 cm in diameter with a white circle of approximately 2 cm diameter in the centre;
- Small plastic toys (e.g., soldiers, frogs) approximately 4–5 cm high.

Prior to the first class, and with the chickens having fasted for five hours, the first author (as handler) took each chicken from its cage and into the room. As soon as the chicken was released on to the table, the clicker was pressed by the second author (as trainer). The clicking sound was immediately followed by offering the food to the chicken (with the cup half full to avoid spillage, which would distract and satiate the chicken, thus slowing the rate of training). Several clicks and treats were given, approximately 3–5 s apart. At this stage, the clicker was not pressed to coincide with any specific behavior of the chicken, as the aim was to habituate the chicken to the new surroundings and feeding equipment (*i.e.*, the cup being presented by a human hand), and build the association (classical conditioning) between the clicker and being fed. After approximately five clicks and treats, the chicken was then picked up and returned to her cage. All chickens successfully pecked from the cup during this initial phase.

A running schedule was used for each class. This included activities with and without the chickens; it was explained to the students that the non-chicken activities were important to allow them to be fast enough to keep up with the pace of the chickens in pecking and learning. Details of how the practical classes were run are included in an Appendix. Each training session was limited to up to five sets of 45 s each. Members of each pair alternated training and being the observer, with each student having up to three opportunities for training within the 2-h practical class.

### 2.3. Questionnaires

A total of 21 items in three sections were included in the questionnaires. The first section consisted of seven demographic questions (e.g., name, age, previous contact with chickens, previous animal training experience). The second section consisted of eight questions relating to chickens and animal training. Each question was answered by circling a Likert-type response from 1 (strongly disagree) to 5 (strongly agree). The questions included:

1. I think that chickens are a difficult animal to train
2. It is easy to teach chickens to do tricks
3. Chickens are intelligent animals
4. Chickens are slow learners
5. Chickens all have individual personalities
6. I feel confident in my ability to train animals
7. I know a lot about training animals
8. I need more practice to be able to train animals effectively

The third section consisted of six questions relating to affective states of the chickens, with the questions asked as “Do you think that most chickens can feel the sensation of…” and the six affective states hunger, pain, fear, boredom, frustration and happiness. Students used a visual analogue scale adapted from Phillips and McCulloch (2005) [13], and placed a cross on a 74 mm dotted line, with “Yes” on the left side and “No, not at all” on the right side of the line. The distance in mm from the left side of the line to the centre of the cross was measured for each response. The post-questionnaire was the same as the pre-questionnaire, except the seven demographic questions were omitted as they had already been answered.

### 2.4. Statistical Analysis

All data were entered into an SPSS 20 (Chicago, IL, USA) spreadsheet, checked for errors, and negatively worded items were reverse coded prior to subsequent analysis. 

Differences between the pre-course and post-course scores were tested using t-tests for continuous data and z tests for categorical data. Significance for all tests was set at *p* < 0.05. Unless indicated otherwise, data are expressed as Mean ± SD. Due to the gender imbalance with many more females than males, the data were weighted to reflect the 50:50 gender balance of the general population.

### 2.5. Ethics

The survey of students was approved by the Human Ethics Committee of the University of Adelaide (H-2013-063). The use of chickens was approved by the Animal Ethics Committee of the University of Adelaide (S-2012-002A).

## 3. Results

There were 122 students enrolled in the course. A total of 102 (83.6%) completed the pre-surveys, with 94 students also completing part or all of the post-survey (92.2%). Unfortunately, eight students were not aware of some questions due to not turning the last page over in the post-survey. The sample was 82% female with an average age of 21 years, and the oldest student 40 years of age (Table 2). Half the number of students had kept chickens, and most of those students had kept 10 or fewer chickens. There were 23 students who had had no previous contact with chickens.

**Table 2:** Demographic data and training history for first year Animal Science and Veterinary Bioscience university students who completed the pre-survey.

Demographic Factors	Number (Range)
Gender (Male:Female)	18:81
Program (Animal Science: Veterinary Bioscience)	29:70
Age (mean ± SD, (range))	21.2 ± 4.1 (17–40)
Have you ever kept chickens?	Number (%)
No	52 (51)
Yes	50 (49)
If yes, how many?	
10 or less	35
12	3
15	1
20	8
30	2
How much previous contact have you had with chickens?	
None	23 (22.5)
Occasional	56 (54.9)
Regular	17 (16.7)
Substantial	6 (5.9)
Have you ever had formal lessons on how animals learn? (*n* = 101)	
Yes	17 (16.8)
No	84 (83.2)
How much animal training have you done before enrolling in this course?	
None	17 (16.7)
Occasional	55 (53.9)
Regular	26 (25.5)
Substantial	4 (3.9)
What species have you previously trained?	
None	13 (12.7)
Dog	59 (57.8)
Horse	2 (2.0)
Chicken	1 (1.0)
Other	1 (1.0)
>1 species	26 (25.5)

A total of 17 students had participated previously in formal lessons on how animals learn, but the majority of students had not. Only four students reported having done substantial animal training in the past, with 26 having done regular animal training and most (54%) only occasional animal training. Over half of students with previous training experience had trained dogs, and 26% trained more than one species previously. The most common combined species trained were the dog and the horse, but a variety of species were listed, including dairy cattle, meerkats, and koalas. Only 13 students reported not having previously trained any species.

In answer to the Likert-type questions, students rated the intelligence and presence of individual personalities in chickens more highly in the post- *versus* the pre-survey (Table 3). The most substantial differences occurred in student attitudes to chickens being difficult to train (21.6% disagreeing or strongly disagreeing in pre-test *versus* 85.1% post-test, *p* < 0.0001) and chickens being slow learners (20.5% disagreeing or strongly disagreeing in pre-test compared to 75.5% post-test, *p* < 0.0001). Most changes for these two variables occurred from the “Neutral” rating in the pre-test to “Disagree” or “Strongly Disagree”. Students were also more likely to agree chickens are easy to teach tricks, have individual personalities and are intelligent in the post- *versus* pre-surveys. Only 7% of students believed they had good knowledge of animal training in the pre-surveys, with significant increases in student ratings for perceived knowledge and confidence to train animals in the post-surveys. There was no significant change in ratings for the item on needing more practice to train animal effectively.

**Table 3:** Change in rating of selected aspects of first year Animal Science and Veterinary Bioscience university student attitudes toward chickens and training from the pre- to the post-surveys following a chicken clicker training class. Values are percentages of students that agreed/strongly agreed or disagreed/strongly disagreed with the statement.

Statements	Pre- (%)	Post- (%)	z	*p* (One Tailed)
I think that chickens are a difficult animal to train ^1^	21.6	85.1	−7.424	<0.0001
It is easy to teach chickens to do tricks ^2^	6.9	60.6	3.804	<0.0001
Chickens are intelligent animals ^2^	49.0	76.6	−3.979	<0.0001
Chickens are slow learners ^1^	25.5	75.5	−5.812	<0.0001
Chickens all have individual personalities ^2^	84.3	94.7	−2.344	0.0095
I feel confident in my ability to train animals ^2^	51.5	67.0	−2.204	0.0138
I know a lot about training animals ^2^	6.9	19.1	−2.55	0.0054
I need more practice to be able to train animals effectively ^2^	93.1	88.3	1.15	0.1251

^1^ Proportion of students disagreeing/strongly disagreeing with the statement; ^2^ Proportion of students agreeing/strongly agreeing with the statement.

**Figure 2:**
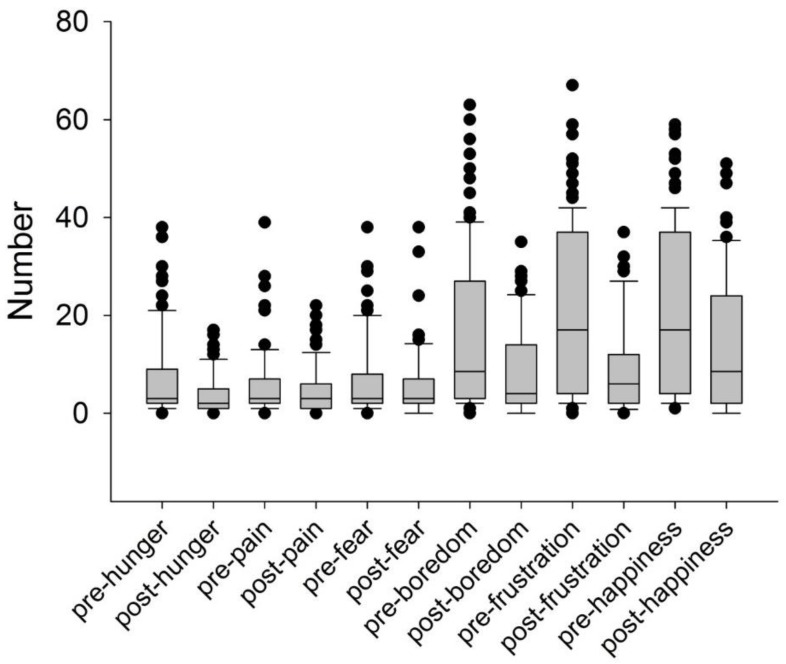
Box plots of pre- and post-scores for first year Animal Science and Veterinary Bioscience university student rating of chickens’ ability to feel affective states using a Visual Analogue Scale (VAS). Values are mm distance from the left edge of the line, with lower numbers representing greater belief chickens can feel each state. Lines in box plots represent the mean value, the box represents the 25th to 75th percentiles and the error bars the 5th to 95th percentiles. Individual outliers are represented by a dot.

In the pre-surveys, students rated the ability of chickens to feel hunger (7.3 ± 8.2 mm), pain (5.3 ± 6.5 mm) and fear (6.7 ± 7.9 mm) more highly than to feel boredom (15.9 ± 16.2 mm), frustration (19.8 ± 16.8 mm) or happiness (20.4 ± 16.9 mm) (Figure 2). Students’ ratings of the ability of chickens to experience affective states significantly changed from the pre- to post-class surveys (Figure 2). Only 66 students (70%) completed this part of the survey post-class. The largest changes in distances from pre- to post-surveys were for boredom (−9.1 ± 15.4 mm, *n* = 65; *p* < 0.001), frustration (−12.1 ± 15.2 mm, *n* = 66; *p* < 0.001) and happiness (−7.8 ± 13.5 mm, 65; *p* < 0.001). There were proportionately smaller changes in hunger (−3.7 ± 7.2 mm, *n* = 66; *p* < 0.001), and no significant change in rating of the ability of chickens to feel pain (−1.4 ± 6.8 mm, *n* = 66; *p* = 0.1) and fear (−1.6 ± 9.0 mm, *n* = 66; *p* = 0.15).

### 3.1. Gender Differences 

Females were significantly more likely than males to agree that chickens are intelligent animals in both the pre- and post- surveys, but there was no difference between males and females for the other ratings (Table 4). There was also no significant difference between genders in agreeing animals have individual personalities, although there was a trend for females being more likely to agree for both pre- and post-surveys.

Females but not males increased their confidence to train animals in the post- *versus* pre- surveys, with females more confident in the post-surveys than males (*p* = 0.03). In the post-surveys, females were less likely than males to disagree that they did not know a lot about training animals (*p* = 0.015), and only the females changed this knowledge rating between the pre- and post-surveys.

Students rated the ability of chickens to feel the affective states on a mm scale, with lower numbers representing great belief that the chicken could experience that state. Only boredom and frustration ratings were significantly different between genders in the pre-surveys, with females more likely to believe chickens could feel boredom (*p* < 0.01) and frustration (*p* = 0.02) than males (Table 5). In the post-surveys females were more likely than males to believe chickens could feel, pain (*p* = 0.01) and fear (*p* = 0.02).

**Table 4:** Male and female students’ attitude to statements about chickens and training animals pre- and post- a practical class clicker training chickens. Values are percentages either agreeing or disagreeing with the statements made.

Statements	Pre-Male %	Pre-Female %	z (p)	Post-Male %	Post-Female %	z (*p* value)
I think that chickens are a difficult animal to train ^1^	17.6	22.4	−0.576 (0.56)	88.2	84.2	−0.365 (0.36)
It is not easy to teach chickens to do tricks ^1^	41.2	32.9	0.783 (0.43)	11.8	10.5	0.283 (0.78)
Chickens are intelligent animals ^2^	17.6	56.8	−3.955 (0.0002)	52.9	81.6	−3.034 (0.002)
Chickens are slow learners ^1^	11.8	30.3	−2.073 (0.038)	70.6	76.3	−0.639 (0.52)
Chickens all have individual personalities ^2,3^	76.5	88.2	−1.601 (0.109)	88.2	96.1	n/a
I feel confident in my ability to train animals ^2^	47.1	54.7	−0.727 (0.467)	47.1	70.7	−2.224 (0.026)
I know a lot about training animals ^1^	64.7	45.3	1.761 (0.078)	52.9	28.0	2.445 (0.015)
I need more practice to be able to train animals effectively ^2,3^	100	92.0	n/a	94.1	89.3	n/a

^1^: Per cent disagreeing or strongly disagreeing on the 5-point Likert scale with the statement; ^2^: Per cent agreeing or strongly agreeing on the 5-point Likert scale with the statement; ^3^: Neither sample satisfies the z test’s standard binomial requirement that n(p) and n(1–p) must both be equal to or greater than 5; n/a: not applicable.

**Table 5:** Male and female students’ attitude of the affective states of chickens pre and post a practical class clicker training chickens using a Visual Analogue Scale (VAS). The numbers represent the distance in mm the student placed a cross from the left side of a line, with agreeing on the left to disagreeing on the right that chickens could experience these affective states.

Mean (SD) mm								
	Pre		t(df)	p (2-tailed)	Post		t(df)	p (2-tailed)
	Male	Female			Male	Female		
Hunger	5.7 (6.0)	7.4 (8.5)	1.1(96)	0.27	4.6 (4.4)	4.0 (4.0)	0.6(69)	0.52
Pain	7.0 (9.9)	5.0 (5.4)	1.3(96)	0.21	7.7 (7.2)	3.9 (3.9)	2.6(69)	0.01
Fear	7.3 (7.6)	6.5 (8.1)	0.5(96)	0.56	9.7 (11.4)	4.6 (4.8)	2.4(69)	0.02
Boredom	25.4 (18.6)	13.5 (14.9)	3.4(93)	0	11.2 (8.9)	7.7 (8.9)	1.7(69)	0.1
Frustration	28.8 (19.9)	17.3 (15.3)	3.2(96)	0	12.8 (11.0)	8.2 (9.2)	1.9(69)	0.07
Happiness	20.7 (16.0)	19.9 (17.2)	0.2(96)	0.82	17.6 (13.9)	12.8 (14.2)	1.4(68)	0.2

There was a larger change from pre- to post-surveys in the mean score for affective states for males *versus* females for boredom (−17.85 ± 15.42 mm *versus* −6.92 ± 14.79 mm, *t* = −2.97, *p* = 0.004, respectively) and frustration (−20.86 ± 19.24 mm *versus* −9.71 ± 13.07 mm, *t* = −2.88, *p* = 0.005, respectively). There was no significant difference between the change in score according to gender for hunger, pain, fear or happiness.

### 3.2. Other Differences 

Whether or not students had previously kept chickens did not significantly affect the changes in ratings of attitudes to chickens and their affective states. There was also no difference in the pre-test surveys between students who had or had not previously kept chickens. Students who had no prior experience in training animals were significantly more likely to improve their self-rating of their knowledge and ability to train animals (M = −0.62, SD = 0.62 for no prior experience; M = −0.1, SD = 0.64 for at least some prior experience); t(90) = −3.2, *p* = 0.002) and their ratings of the extent to which chickens feel pain (M = −5.81, SD = 11.3 for no prior experience, M = 0.3, SD = 7.1 for at least some training experience; t(68) = −2.63, *p* = 0.011), but not any other item. Student age was a significant factor, with older students less likely to rate chickens as intelligent animals (r^2^ = −0.21, *p* = 0.043); less confident in their ability to train animals (r^2^ = −0.25, *p* = 0.016); and more likely to believe they needed more practice to train effectively (r^2^ = 0.31, *p* = 0.003). There were no relationships with student age and change in attitudes toward other abilities and affective states of chickens.

### 3.3. Informal Written Feedback

Students’ written informal feedback on the class suggested they had underestimated how difficult it was to clicker train a chicken:

*“It was a lot more difficult than I expected to decide what behaviour deserved a click and which didn’t…”*

Another student commented:

“I learnt I need to improve motor skills to be faster than (the) chicken.”

Written comments made by students in their practical notes also related to a shift in their attitudes, particularly towards the intelligence of chickens. For example, students wrote:

“Interesting that chickens are a lot smarter than I originally thought.”

“I never thought that chickens would be intelligent enough and learn quite so quickly.”

“I did not know chickens could learn quite fast.”

## 4. Discussion

This study has demonstrated for the first time substantial shifts in student attitudes to chickens and their beliefs in whether or not they experience affective states (e.g., boredom, happiness) following a practical class in which students learnt to clicker train chickens. Thus, practical classes in veterinary and animal science programs can not only provide students with important skills, but also may influence their attitudes to the species with which they are working.

Belief in animal mind is a term used for the attribution of mental capacities, such as intellect, ability to reason, and feelings of emotion, to animals [2]. The data confirm that a greater knowledge of the cognitive abilities of an animal may lead to more positive attitudes to that animal. Background knowledge of chickens and their abilities was relatively low in the cohort, with 23 students having no previous contact with chickens. Associations picked up through the media are likely to be images of chickens in their hundreds if not thousands in the media in battery cage or broiler production, which may encourage a perception they are not individuals and do not display complex behaviors. Despite this popular perception, the majority of the students in this study agreed with the statement that “Chickens all have individual personalities”, even in the pre-test.

Student attitudes to the capacity of chickens to feel the different affective states varied both in terms of which states most students believed chickens could feel, and also between students. It was interesting that most students agreed that chickens could feel hunger, pain and fear, but were less likely to believe chickens could feel boredom, frustration and happiness in the pre-surveys. However, boredom, frustration and happiness were the affective states with the greatest shifts in student attitudes in the post-surveys. Sentience is a concept which includes the ability of an animal to feel positive and negative emotions and pain. In studies of students from different countries, students rated the ability of various animals according to the ability to feel pain, happiness, fear and boredom [13,14]. Unfortunately, the individual scores for each sensation were not presented, but the scores were combined to form an overall level of sentience *versus* adult humans. The level of sentience in chickens was rated below the monkey, dog, fox and pig, but above the rat and fish [13,14]. In the second study, chickens rated lower than the chimpanzee, dog, dolphin, cat, horse, cattle pig and rat, but above the octopus and fish [14]. These studies demonstrate the baseline level of sentience attributed to chickens is relatively low compared with other common domestic species. 

While there were changes in the attitudes of the students to chickens, it is not certain if these would translate to differences in behavior in areas such as choosing free range over battery cage produced eggs, or consumption of chicken meat. Some philosophers claim that sentience is the main reason for attributing “interests” or a sense of moral worth to animals [15,16]. Thus, beliefs about the level of sentience of an animal may be likely to translate to changes in their moral worth. Students who attributed higher levels of sentience to chickens were less likely to agree with the statement “As long as adequate food, warmth and light are provided, there is nothing really cruel about battery hen farming” [13]. In a review paper, Serpell [1] suggested science can play a role in facilitating positive attitudes to animals and reducing negative ones. The present study suggests exposing people to the cognitive abilities of a specific species may promote more positive attitudes towards that species. This is particularly interesting with a shift towards a promotion of positive states to ensure “good” animal welfare, rather than “good” welfare simply being the absence of negative states [17]. Promotion of positive welfare in a specific species is likely to work better if people do believe a species is capable of experiencing positive affective states, otherwise they might not bother.

Females tended to have a more positive view of chickens’ abilities than males did before the chicken clicker training workshop, and their experiences, as shown by their post- survey ratings, seemed to strengthen their existing views. However, while males were more likely to be neutral or negative in their attitudes toward chickens, the clicker training workshop still served to significantly change their opinions. In a previous study with an earlier cohort participating in the same course, attitudes to animals in females were more positive and changed more following the course than for males [18]. There was no chicken clicker training practical class included in the course at that stage. Females have had a higher score, representing a more positive attitude to animals, in other studies using surveys to compare genders [19,20].

As well as a gender difference in attitudes to the chickens, there was also a gender difference in how the practical class improved confidence in their ability to train other animals, with a more positive change in females. Males have generally been found to rate themselves more highly in competence to perform various practical tasks than females [21,22,23]. This study would suggest that following time to learn and practice their skills, females may improve in their confidence to train animals compared to males. However, the sample size for males was much smaller than for females, and these results require future confirmation.

Practical knowledge includes skills or “knowing-how” to do things [24]. Practical skills develop in the specific situation in which they are taught, and are both contextual and social in nature [25]. Although providing practical experience in training animals is an important adjunct to theoretical learning, there has been only one published report on teaching students to use clicker training [5]. In the present class, there were some basic mistakes made by students that made it clear they did not implicitly understand the theoretical knowledge of training at a practical level. For example, one student persisted in clicking and not feeding the chicken, and needed to be corrected. 

As well as the benefit of students understanding how animals learn, the practical class also encouraged the use of positive reinforcement training techniques. In recent years, there has been a shift from punishment based training techniques to those based on positive reinforcement with studies finding the latter to be more effective [26,27]. Punishment based training has been associated with an increased risk of dog aggression to both humans and to other dogs [3,4]. Use of a clicker as a bridging stimulus can provide an even clearer signal to an animal on the specific behavior being rewarded, since the click is timed to coincide with that behavior. The clicker may also provide an enhanced effect on learning, as the click ends the behavior, which in turn gives the animal the opportunity to repeat the behavior (and be reinforced) many times within a short space of time.

Due to timetabling limitations, students were all at the same stage of the course when they completed the pre-surveys, but completed the post-surveys between Weeks 1 and 8 of the semester. This meant in the first two weeks students had not covered learning theory prior to the practical class, and in later classes students had also learnt more about animal cognition, animal welfare and animal ethics, which may also have changed their attitudes to chickens. In a previous study, we showed that following participation in the same course in 2008 (without the chicken clicker training practical class), veterinary but not animal science students had a significantly more positive attitude towards “profit” animals using a questionnaire evaluating attitudes to Pet, Pest and Profit animals separately [18]. Any teaching class that increases positive attitudes to animals may be particularly valuable in a veterinary program, particularly as male students’ empathy to animals and belief in animal sentience may decrease during their study [28]. When changes in the survey questions were plotted against the week that the students participated in the practical class, there was no obvious pattern according to the week, although the numbers of students may have been too small to see an effect. A further limitation of the study was that there were fewer males than females, with low numbers of males completing the post-surveys. Therefore, conclusions on gender differences need to be verified using a larger sample size. Finally, the intervention was relatively short, and further study is necessary to determine if any longer term changes in attitude or behavior occur following a chicken clicker training workshop.

## 5. Conclusions

A practical class to teach animal training skills was effectively implemented in an undergraduate course for animal science and veterinary students. Following participation in the class, significant changes in student attitudes to chickens were found using questions relating to chicken intelligence, personality and ability to experience affective states. Such classes may be beneficial in improving attitudes towards the level of sentience of chickens or other animals. Future work is needed to demonstrate if these changes in attitudes translate to changes in behavior, such as purchasing free range *versus* battery cage eggs. Just as importantly, student understanding and practical knowledge of animal training may translate to more effective positive reinforcement based methods, and improved animal welfare.

## Appendix

### Procedure Used for Running a Chicken Clicker Training Practical Class

The schedule is detailed below:

1. Introduction to clicker training and chickens
2. Random allocation of pairs of students to one chicken each
3. Mechanical skills exercise 1—the “home” position with the cup held against the trainer’s abdomen between the navel and rib cage and covered with the other hand was demonstrated. Students then practiced clicking and feeding by quickly placing the cup (initially empty, and then half filled with pellets) without banging it, on the table over the target and returning to the “home” position. This was demonstrated first by the authors. Pairs then practiced in 45 s sets, allowing for approximately 30–40 repetitions per set, with each member alternating between observing and coaching the other on minimizing body movement and returning the cup to the home position in each set.
4. One member of the pair brought out their chicken, placed her on the table and the other member of the pair clicked and fed from the plastic cup, allowing approximately 5 pecks of food.
5. Mechanical skills exercise 2: One member placed individual playing cards on the table as quickly as possible and the other member clicked when they saw the card type called out by the demonstrator (e.g., red cards, even number cards).
6. Chickens were again brought to the table, clicked and fed with the member of the pair changing roles.
7. Teaching “shaping” in pairs—“shaping” means rewarding of a task (e.g., placing hands on head) that approximates the final task required e.g., placing hands on head, standing on one foot, or picking up one of the toys and placing it in pocket, using only the clicker to mark successive approximations toward the goal behaviour. This was first demonstrated by a volunteer with the instructor being the trainer, then pairs of students worked together to train each other. The students were asked to write the goal behaviour down before beginning the training, so that the authors could see what the behaviour in progress was aiming toward, observe the trainer and offer coaching.
8. Begin shaping of chickens to peck on a target (the coloured circle). Note that the activity trained varied according to the week of the practical class, in the first two weeks chickens were trained to peck on the target, but in subsequent weeks the task varied according to what the chicken had already learnt. For example, chickens that pecked on red circles in the first two weeks were taught colour discrimination by being presented with both a green and a red target, and only clicked and treated for pecks on the red target. Detailed notes on what each chicken learnt in the class were written by each pair, and these were entered into a spreadsheet so that in each subsequent week the authors knew what each individual chicken had already learnt.

Each training session was limited to up to five sets of 45 s each, and at the end of each training session students who were excited about having trained a task or who were having problems were invited to give a demonstration to the rest of the class. Feedback was then given by the authors and other students on what the trainer was doing well, and what could be improved. Training sessions were broken up by discussions and students filling out written notes. These breaks enabled the chickens to have access to water between training sessions and slowed down the rate of satiation. Members of each pair alternated training and being the observer, with each student having up to three opportunities for training within the 2-h practical class.
